# miRNome Characterization of Milk-Derived Extracellular Vesicles in Recombinant Somatotropin-Treated Dairy Cows

**DOI:** 10.3390/ijms26062437

**Published:** 2025-03-08

**Authors:** Alessandro Benedetto, Nunzia Giaccio, Maddalena Arigoni, Raffaele Adolfo Calogero, Patricia Regal, Alexandre Lamas, Francesca Martucci, Valentina Audino, Gaud Dervilly, Marzia Pezzolato, Elena Bozzetta

**Affiliations:** 1 Istituto Zooprofilattico Sperimentale del Piemonte, Liguria e Valle d’Aosta, Via Bologna 148, 10154 Torino, Italy; nunzia.giaccio@izsplv.it (N.G.); francesca.martucci@izsplv.it (F.M.); valentina.audino@izsplv.it (V.A.); marzia.pezzolato@izsplv.it (M.P.); elena.bozzetta@izsplv.it (E.B.); 2 Dipartimento di Biotecnologie e Scienze della Salute, Core-Lab di Bioinformatica e Genomica, Università degli Studi di Torino, 10124 Turin, Italy; maddalena.arigoni@unito.it (M.A.); raffaele.calogero@unito.it (R.A.C.); 3 Food Hygiene, Inspection and Control Laboratory (LHICA-USC), Department of Analytical Chemistry, Nutrition and Bromatology, Faculty of Veterinary Science, Universidade de Santiago de Compostela, 27002 Lugo, Spain; patricia.regal@usc.es (P.R.); alexandre.lamas@usc.es (A.L.); 4 Oniris, INRAE, LABERCA, 44300 Nantes, France; gaud.dervilly@oniris-nantes.fr

**Keywords:** milk, miRNA, recombinant somatotropin, extracellular vesicles, untargeted transcriptomics, biomarker

## Abstract

The recombinant bovine somatotropin (rbST) is a synthetic hormone developed to mimic the effects of the endogenous growth hormone, also known as bovine somatotropin (bST). Although rbST use in dairy cows is authorized in several countries, it is currently banned in Europe. Different methods for screening and confirmatory detection of rbST were developed, mainly based on LC-MS/MS and immune-enzymatic assays. However, some commercial forms of rbST have above the same amino acid sequence of bST, making it difficult to produce a reliable differentiation of recombinant from endogenous forms. Complementary strategies for indirect detection of rbST can therefore be considered as alternative biomarker-based tools. Untargeted transcriptomics was applied to characterize the microRNAs (miRNA) isolated from milk extracellular vesicles (EVs) in rbST-treated animals, aiming the identification of non-coding biomarkers related to its administration. Sequencing analysis of 63 archive samples collected during previous animal trial allowed for the identification of 35 differentially expressed (DE) miRNAs. A validation study performed by qPCR on a further 70 milk samples from a field survey confirmed the significant upregulation of bta-miR-10167-3p in milk EV from rbST-treated cows. The results obtained suggest the potential use of bta-miR-10167-3p as a non-invasive biomarker to be considered in novel screening strategies, needed to tackle rbST misuse in dairy cows.

## 1. Introduction

Bovine growth hormone (bGH) or somatotropin (bST) is a peptide hormone secreted by the anterior pituitary gland, with a primary sequence of 190–191 amino acids, corresponding to a molecular weight (Mw) of 22 kDa. Once bST is released through the bloodstream, it can reach and bind to liver receptors, activating different signaling pathways and promoting the release of insulin-like growth factor (IGF-1), one of the main modulators of bST growth effects. Indeed, bST affects metabolism, body composition, and cardiovascular function, influencing not only muscle strength, but also other physiological processes related to cellular regeneration [1].

The first correlations between growth hormone administration and galactopoiesis in dairy cows came from studies on pituitary gland extracts administered to cattle [2]. Several investigations then confirmed, among different somatotropin effects, a significant increase in milk yield [3,4] achieved by the delaying mammary glands involution and enhancing the maintenance of secretory cells, thereby extending milk production beyond the peak lactation. Subsequently, with the advent of biotechnology applications based on DNA technology and genetic engineering, large-scale synthesis of recombinant bovine somatotropin (rbST) became possible. Extensive studies on dairy cows concluded that administering rbST or bST before peak production extended lactation [5].

Recombinant bovine growth hormone is currently produced in different formulations that could differ from the primary endogenous variant, with, for example, a methionine (Met-rbGH) in substitution of the alanine (Ala) in the N-terminal position, while others have an amino acid sequence identical to that of the main endogenous variants (Ala-rbST) [6].

Despite the approval of rbST for zootechnical purposes in many countries [7], in 1999, the European Union (EU) banned treatments with this hormone in livestock following the Decision 1999/879/CE [8], and it listed the rbST among the protein hormones group (A3e) in Regulation 2022/1644 [9]. This ban was based on concerns related to animal welfare, potential impacts on European milk production policies and, lastly, on consumer health.

Since then, several EU investigation agencies have reported cases of illicit use of rbST, as a result of black-market activities and unauthorized drugs imports from non-European countries, undermining fair trade and the law prescriptions [10].

For this reason, efficient and reliable analytical techniques are required to monitor rbST misuse, according to Commission Implementing Regulation 2021/808 and Commission Regulation 2017/625 [11,12].

Among the proposed strategies against rbST misuse, the detection of anti-rbST antibodies, induced and released in blood and milk of dairy cows after repeated drug injections to maintain their milk production, seems to be one of the most promising biomarker-based test for screening purpose [13,14]. Then, at the confirmatory level, different liquid chromatography-mass spectrometry (LC-MS/MS) methods for detection of rbST in bodily fluids were developed with the aim to provide absolute proof of the identity of the compound of abuse [15,16,17,18,19].

However, as recently reported by the European Reference Laboratory (EURL) for growth promoting compounds, available methods (both screening and confirmatory) still have limitations that could affect their application in field as reliable tools for competent authorities in charge of drug residues control plans management [20].

Therefore, novel approaches for both direct (residue-based) and indirect (biomarker-based) detection of rbST abuse are needed, ideally focusing on easily collectable milk samples. This would avoid the need for blood/serum sampling from individual cows and allow the testing of all dairy cows at once, whether by individual or bulk milk sampling. To meet the requirements of official control plans in the EU, molecular-based methods have been proposed for rbST detection. In this context, the growing application of high-throughput techniques like metabolomics, proteomics, and transcriptomics could surely help to capture different sets of molecular alterations caused by exposure to growth promotors [21].

Among different proposed omics characterizations, transcriptomic profiling of milk somatic cells and other easily accessible/collectable specimens (e.g., hair follicles), have provided promising preliminary results [22,23], highlighting the potential diagnostic application of rbST transcriptional biomarkers for official control purposes.

However, given the low recovery of coding RNA from milk somatic cells (that are usually expected to be low in healthy cows), the characterization of more abundant RNA species in milk, such as microRNAs (miRNAs), could potentially allow the identification of additional transcriptomics signatures related to rbST treatment in dairy cows.

MicroRNAs (miRNAs) are indeed small gene-regulatory non-coding RNAs that are highly enriched in cow milk. They are enclosed in extracellular vesicles (EVs) that protect them from the environment and the adverse conditions of the gastrointestinal tract during milk consumption and digestion [24], preserving their epigenetic potential towards the receiving organism.

These distinguishing features of miRNAs contained and conveyed via EVs, was further assessed by several in vitro studies, revealing how cow milk EVs may transfer miRNA cargos to human cells and regulate recipient cells gene expression similarly to endogenous miRNA expressed in the host, or received by human breast milk during postnatal period [25,26].

The aim of the work was, therefore, to characterize the non-coding transcriptome of milk-derived EVs collected from rbST-treated dairy cows, enabling the detection of miRNAs exploitable as potential biomarkers of recombinant somatotropin treatment for field investigations.

## 2. Results

### 2.1. Sample Selection and Quality Assessment

**Figure 1:**
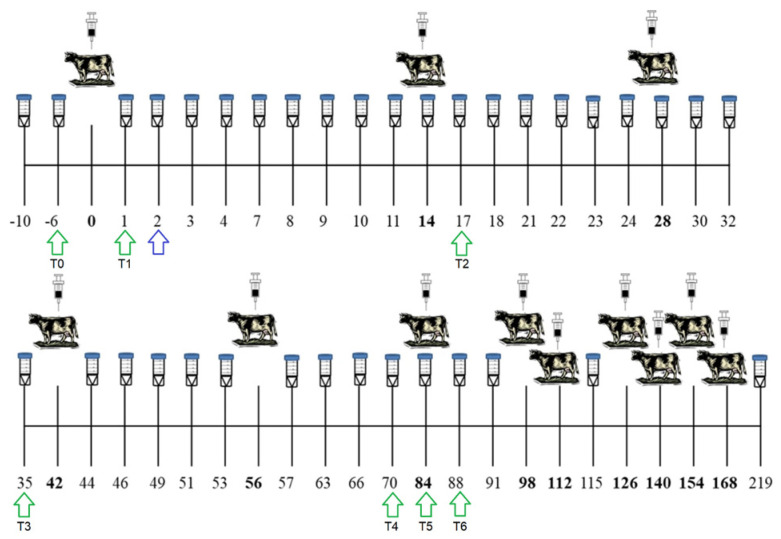
Visual representation of rbST time course administration during animal trial (modified from the original work of Lamas and colleagues [23]). The number 0 refers to the day of the first injection (−6 refers to six days before the first rbST administration, etc.). Green arrows represent the sampling point available for miRNA sequencing analysis (T0–T6). Blue arrow represents additional sampling of a single animal from the treated group two days after the first rbST injection.

Sixty-four archival milk samples (years 2016–2017), originally collected from nine animals enrolled in a long-term exposure experiment performed in Spain [23] to study rbST treatment (Figure 1), were selected—being still available from the biobank of the Faculty of Veterinary Science, Universidade de Santiago de Compostela (USC)—and processed together with seventy field milk samples (years 2022–2023) collected from six dairy cows during a survey carried out in Italian farms located in the Piedmont region (north-western Italy). Both experimental and field animals were Holstein/Friesian dairy cows. This choice was made not only to limit potential confounding variance related to different breeds, but also by considering that Holstein/Friesian breed accounts for almost 95% of the herd animals in Europe [27], producing most milk that is consumed in the European Union (EU).

All collected samples were submitted to preliminary evaluation by different approaches prior to sequencing and qPCR analysis, as reported in Section 4: NTA tracking, analysis of milk quality, milk composition, and RNA recovery.

In depth, all archival samples, stored at −20 °C for more than 6 years, still showed acceptable profiles for both EVs size distribution and concentration but, as expected, the highest vesicles concentrations and lowest SD in EV width distributions were recorded for the more recent field samples (see Supplementary Material S1). However, mean and mode values of vesicles sizes distributions and following miRNA sequencing outputs (see Supplementary Material S1 and Table 1 in Section 2.2) were in line with the data presented in previous studies (see Section 3).

Regarding field survey in Italian local farms, during the last months of sampling activities, one of the selected cows went dry and was therefore replaced by another dairy cow from the same farm. All tested milk quality parameters from these samples, collected during field survey, resulted in line with fixed threshold standards for cow milk required by current legislation (see Supplementary Material S2), confirming the mastitis-free status of all animals enrolled in the study (no clinical symptoms and milk somatic cell counts always below 100,000 cells mL^−1^). Same results on milk quality parameters were originally recorded on samples from rbST exposure study [22,23,28].

RNA concentrations from isolated milk EVs ranged between 1.3 ng/uL and 5.2 ng/uL in archival samples and from 9.1 ng/uL to 25 ng/uL in field samples, respectively.

### 2.2. Small RNA Sequencing

Raw sequencing results are summarized in Table 1. After basic quality controls (QC), only one sample (s2T1t) from a control cow belonging to sampling point T1 (1 day after first rbST dose on the treated group) resulted under the threshold of 10K QC-passing reads mapped to *Bos taurus* miRNA precursors, and therefore it was removed from the study. All collected sequencing data are available under the BioProject accession number PRJNA1145299 in the NCBI repository.

**Table 1:** Total sequencing output and miRbase mapped reads for the 63 milk samples analyzed in the study, referred to each cow (Animal ID 1–9), their relative status (control or rbST-treated animal), and sampling time (from T0 to T6, as already shown in Figure 1). Only one sample (S2T1t) resulted below the 10k reads mapped to reference transcriptome.

Sample_ID	Total Seq	Mapped	rbST	Sampling Time	Animal ID
s1T0c	3,828,519	208,063	control	T0	1 *
s1T1t	3,085,517	122,773	treated	T1	1
s1T2t	4,316,225	471,009	treated	T2	1
s1T3t	29,983,329	2,695,040	treated	T3	1
s1T4t	9,019,457	1,050,819	treated	T4	1
s1T5t	2,869,470	153,915	treated	T5	1
s1T6t	10,494,740	1,473,190	treated	T6	1
s2T0c	4,796,779	105,297	control	T0	2 *
s2T1t	188,968	6997	treated	T1	2
s2T2t	9,381,548	1,005,499	treated	T2	2
s2T3t	22,133,967	2,934,448	treated	T3	2
s2T4t	30,356,677	1,647,234	treated	T4	2
s2T5t	8,165,814	571,127	treated	T5	2
s2T6t	7,895,100	1,421,498	treated	T6	2
s3T0c	4,105,600	153,699	control	T0	3 *
s3T1t	4,238,014	129,415	treated	T1	3
s3T2t	5,682,647	598,925	treated	T2	3
s3T3t	15,537,934	2,187,094	treated	T3	3
s3T4t	11,499,701	1,765,815	treated	T4	3
s3T5t	7,786,629	357,732	treated	T5	3
s3T6t	13,280,173	1,449,837	treated	T6	3
s4T0c	2,303,125	83,933	control	T0	4 *
s4T1t	5,577,948	149,493	treated	T1	4
s4T2t	5,504,047	495,298	treated	T2	4
s4T3t	33,127,593	3,162,873	treated	T3	4
s4T4t	14,886,381	1,353,519	treated	T4	4
s4T5t	11,285,114	1,445,212	treated	T5	4
s4T6t	10,188,989	1,588,432	treated	T6	4
s5T0c	2,671,228	56,547	control	T0	5 *
s5T1t	4,437,871	158,497	treated	T1	5
s5T2t	5,712,065	655,868	treated	T2	5
s5T3t	38,259,506	3,205,486	treated	T3	5
s5T4t	8,952,112	904,927	treated	T4	5
s5T5t	9,481,162	507,158	treated	T5	5
s5T6t	1,056,805	78,917	treated	T6	5
s6T0c	5,337,415	304,115	control	T0	6
s6T1c	3,868,419	242,346	control	T1	6
s6T2c	9,156,540	987,353	control	T2	6
s6T3c	24,636,864	4,299,933	control	T3	6
s6T4c	8,479,445	1,117,713	control	T4	6
s6T5c	33,312,826	697,921	control	T5	6
s6T6c	21,510,851	4,221,042	control	T6	6
s7T0c	7,458,017	669,561	control	T0	7 *
s7T1t	5,744,949	357,546	treated	T1	7
s7T2t	23,118,617	2,514,111	treated	T2	7
s7T3t	23,170,624	2,014,974	treated	T3	7
s7T4t	17,362,628	2,029,080	treated	T4	7
s7T5t	20,218,176	3,189,136	treated	T5	7
s7T6t	25,071,924	3,863,181	treated	T6	7
s8T0c	2,995,090	92,222	control	T0	8
s8T1c	4,362,023	83,093	control	T1	8
s8T2c	7,924,546	362,089	control	T2	8
s8T3c	3,860,580	304,426	control	T3	8
s8T4c	4,349,526	246,852	control	T4	8
s8T5c	8,111,221	867,770	control	T5	8
s8T6c	4,994,429	593,879	control	T6	8
s9T0c	4,247,464	182,306	control	T0	9
s9T1c	5,136,754	123,945	control	T1	9
s9T2c	10,299,719	668,682	control	T2	9
s9T3c	20,247,505	1,402,433	control	T3	9
s9T4c	9,949,579	861,363	control	T4	9
s9T5c	10,247,184	1,266,658	control	T5	9
s9T6c	5,161,508	805,228	control	T6	9

* Samples from cows belonging to treated group but collected before the first rbST injection (T0). They are, therefore, considered as not treated with rbST (and merged in the gene expression studies with other untreated control samples).

**Figure 2:**
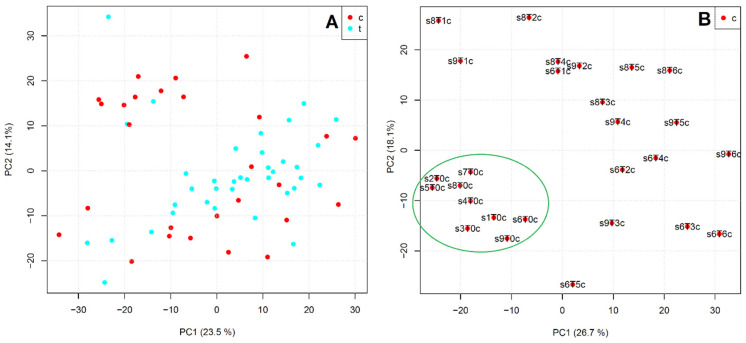
Principal component analysis (PCA) built with relative counting outputs from sequencing data of milk samples collected from experimental animals, considering all rbST-treated (t, blue dots) and control (c, red dots) samples (**A**), and only control samples (**B**).

With the extraction of mapping miRNA reads and relative counting outputs from sequencing data, a data matrix was generated for all samples and then used to perform an exploratory PCA (Figure 2).

No clear separations were recorded when all rbST-treated and control samples were analyzed together (Figure 2A). However, when PCA analysis was limited to only control samples, the clustering of all nine samples collected at T0 was recorded (see green circle in Figure 2B). On the contrary, no clear separations on the two first PC were recorded by narrowing the PCA to only rbST-treated samples.

**Figure 3:**
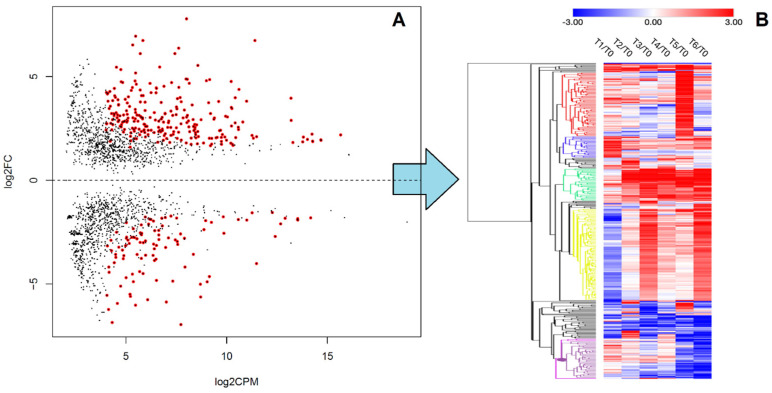
(**A**): Identification of DE-miRNA (red dots) based on ANOVA-like function (edgeR) by comparison of miRNA profiles from all untreated control samples from six sampling points (from T1 to T6) vs. all animals specimens collected at T0 (nine milk samples collected 6 days before first rbST dose, i.e., when all animals were untreated). The analysis globally identified 353 DE-miRNAs. (**B**): Heatmap of the DE-miRNAs list. Five different miRNA clusters (red, blue, green, yellow, and purple) were identified among DE-miRNAs according to applied hierarchical clustering.

For the identification of DE miRNAs related to physiological variations not belonging to rbST treatment (for example circadian rhythm, different stages of lactation from galactopoiesis to involution, etc.), the Anova-like analysis (edgeR) identified 353 DE-miRNAs (Figure 3A).

The hierarchical clustering of the group’s average differential expression (Euclidean distance, average linkage, row clustering) define 5 main clusters as shown in Figure 3B. The miRNA lists of each cluster is further detailed as Supplementary Material S3.

**Figure 4:**
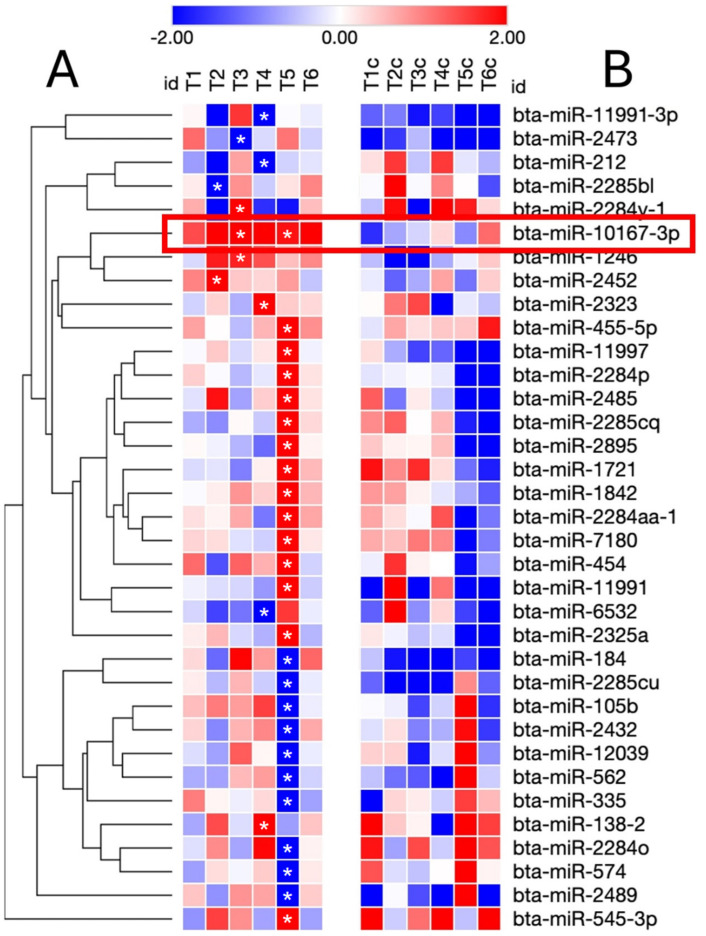
(**A**): Heatmap and hierarchical clustering of the 35 DE-miRNAs identified by small RNA sequencing analysis on milk EVs isolated from rbST-treated cows (T1–T6). (**B**): The collected miRNA profiles were compared with corresponding control cows groups (T1c–T6c). Statistical significance of differential expression (*p* < 0.05) is reported in the figure with the * sign. The Bta-miR-10167-3p shown an upregulation trend in all considered sampling times (see fold changes in red box).

After this preliminary filtering stage of miRNAs resulted differentially expressed within the different control groups considered in the time course study, paired comparisons of each rbST-treated groups versus relative untreated groups at the same time point (i.e., from T1 to T6) were made by the DESeq2 package. The analysis detected globally 35 DE-miRNAs. A hierarchical clustering using the T1 to T6 log2FC is shown in Figure 4, including the behavior of each miRNAs in respective control groups during the six different sampling times analyzed. Among the 35 DE-miRNAs identified by this approach, bta-miR-10167-3p resulted in general always upmodulated in rbST groups.

### 2.3. qPCR Validation Study

**Figure 5:**
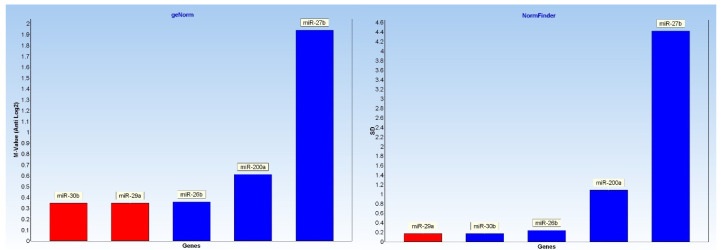
geNorm (**left**) and NormFinder (**right**) stability study. The bta-miR-29a (red bar in right plot) resulted the miRNA with the most stable expression across different field and experimental samples groups.

Analysis on a panel of reference miRNA (see Table 1), needed for proper gene expression data normalization, found bta-miR-29a and bta-miR-30b as the most stable target in both field and experimental milk samples, according to GeNorm and NormFinder algorithms (Figure 5).

**Figure 6:**
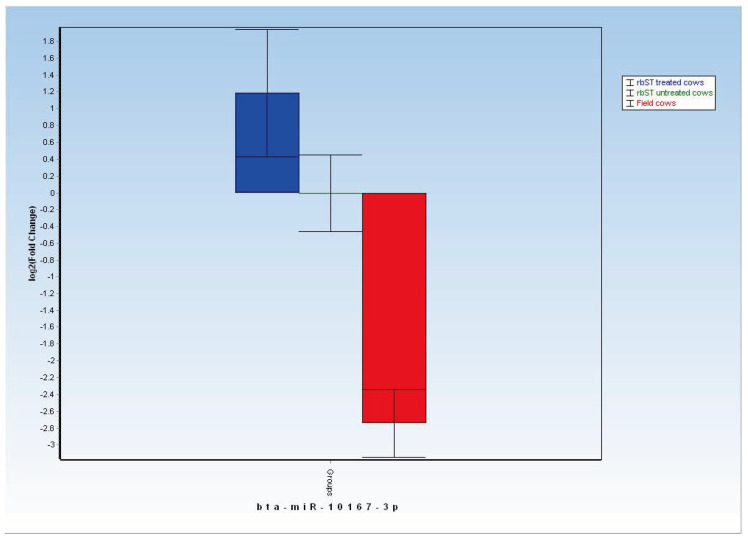
Bta-miR-10167-3p expression levels (fold change (FC) in log2 scale, ±CI at 95%) in milk EV from rbST-treated cows (blue bar on the left, FC = 1.19 ± 0.85), rbST-untreated control cows (central green line, FC = 0 ± 0.45), and field cows (red bar on the right, FC = −2.74 ± 0.4).

Therefore, according to preliminary sequencing analysis, the bta-miR10167-3p was chosen among other DE-miRNAs found for a larger field survey, being systematically up-regulated in all samples collected from rbST-treated dairy cows (Figure 4). The validation of its expression profiles in milk samples from field dairy cows was achieved through qPCR analysis. Specifically, relative quantification analysis on both experimental samples and field samples is reported in Figure 6, confirming significant upregulation of bta-miR10167-3p in milk samples from rbST-treated cows not only when compared to untreated control cows (*p* < 0.05), but also when compared with expression levels recorded in field dairy cows (*p* < 0.001).

**Figure 7:**
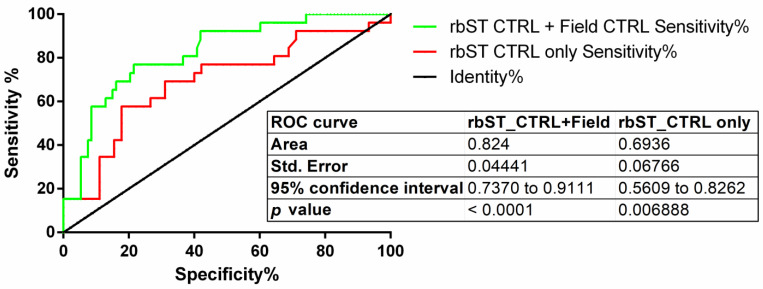
ROC curves to study diagnostic potential of bta-miR-10167-3p relative expression for detection of rbST abuse in dairy cattle.

Receiver operating characteristic (ROC) curve analysis was then performed on bta-miR-10167-3p expression profiles, considering only data collected from experimental animal trial (i.e., rbST-untreated dairy cows milk samples vs. rbST-treated dairy cows milk samples) or, alternatively, by merging both experimental and field samples data together (Figure 7).

When only untreated control animals from the rbST trial were considered, the ROC curve calculations report, with a cut-off of 1.534, a sensitivity and specificity, respectively, of 57.7% (range from 36.43% to 76.65%, 95% CI) and 91.4% (from 83.75% to 96.21%, 95% CI). When also field samples were merged with untreated control animals from the rbST trial, for the tested miRNA, the ROC curve found an increased sensitivity of 76.92%, (ranges from 56.35% to 91.03%, 95% CI) and a specificity of 78.49% (ranges from 68.76% to 86.34%, 95% CI) by lowering cut-off value to 0.0013.

## 3. Discussion

The bovine somatotropin (bST) in dairy cows is known for its role in the stimulation of mammary gland growth and regulation of milk production. Because of these peculiar features, the exogenous administration of bST has been proposed since the 1980’s to increase milk production, especially with the development of recombinant pharmaceutical forms that offer the possibility of large-scale production. The two mains commercially available forms nowadays are Posilac^®^ (Monsanto) and BoostinS-^®^ (Hilac, LG Life sciences). The former is chemically almost identical to the endogenous form, with only a distinct N-terminal methionine (actually the same N-terminal modification of the rbST contained in Lactotropina^®^ form Elanco) used in the animal trial by Lamas and colleagues [23], while the latter is strictly identical to the endogenous bST. Data from previous studies, following recommended treatment schedule in dairy cows, revealed that rbST blood concentrations can be lower than 10 ng mL-1, and even lower in milk [15,18,28], requiring high performance analytical methods based on mass spectrometry for reliable residues quantification.

Within the EU, rbST has been banned since 2000, according to Decision 1999/879/EC. The rbST ban required, therefore, methods for official control purposes aimed to correctly identify and quantify rbST misuse in final food products. Direct rbST detection is complex due to the similarity of recombinant forms with the endogenous hormone (bST) and for the strong fluctuations of bST in serum. Currently available screening methods are focused on detecting rbST-dependent biomarkers, instead of rbST itself. This approach is mainly motivated by the fact that rbST-dependent biomarkers have a longer half-life, offering a promising alternative, similar to what has been reported in the literature for steroid abuse and doping control [29,30,31].

Among different biomarkers categories (transcripts, proteins, metabolites) proposed for indirect monitoring of dairy cows’ exposure to a broad range of drugs and environmental contaminants, profiling miRNas in milk EVs is currently considered one of the most promising strategies, useful to characterize and monitor the physiological and/or pathological status of the mammary gland [32,33,34]. For this reason, microRNAs could potentially reveal the abuse of illicit drugs like rbST, that is known to exert its biological effects by over-triggering the targets and pathways regulated by endogenous bST.

To detect potential rbST-related miRNA markers in milk, a preliminary evaluation of sample quality from available biological material was mandatory. The suitability of archival milk samples collected during a previous animal trial on rbST administration in dairy cows [23] was checked by EVs isolation from available skimmed milk samples, followed by nanoparticle tracking analysis (NTA) before proceeding with RNA extraction, purification, and sequencing analysis.

The effects of prolonged storage on archival samples had indeed some effects on the size profiles and the amount of isolated EVs (Supplementary Material S1), leading to lower recoveries of total RNA from experimental specimens compared to field ones (see Section 2). However, this aspect, as shown in a previous study [35], did not critically impact RNA quality, allowing us to proceed with miRNA profiling by sequencing analysis followed by validation of one of the most relevant DE-miRNA biomarkers by qPCR, and finally by testing field samples. To identify specific miRNA markers dysregulated by rbST administration, a preliminary filtering of all DE-miRNAs detected by sequencing analysis was necessary. Many of these small RNAs are indeed related, as expected, to known sources of physiological variability during the entire lactation period. Actually, several studies confirmed the dynamic nature of milk composition also when considering EV-derived miRNAs: for example, several miRNA patterns in milk EVs have been described as related to immune response modulation [24,36], or associated to different lactation stages [37] influencing cell proliferation [38], as well to somatic cells fluctuations somatic cell fluctuations, circadian rhythm variations [39] and, last but not least, also related to expected interindividual variability influenced by diet and animal management [40].

In depth, Anova-like analysis on all available sampling times (T1 → T6) of the control groups compared to T0 sampling time (when also the animals to be managed according to the rbST schedule were still not treated) allowed for the identification of 353 DE-miRNAs. The following clustering analysis identify five miRNA clusters detailed in Supplementary Material S3. Interestingly, several miRNAs belonging to reported clusters were already described in previous studies as related to early and middle stages of bovine lactation cycle [41], while some other DE-miRNAs like bta-miR-19a, bta-miR-30a-5p, and various bta-miR-2284 families found in our T4, T5, and T6 sampling points (collected in August 2016 during the original animal trial) are previously described as related to summer heat stress in Holstein dairy cows [42]. Actually, high temperatures recorded during the summer of 2016 in the open stables where experimental cows were housed (personal communication from Lamas et al.) may have influenced miRNA profiles recorded within control groups. The following differential analysis (DeSeq2) of rbST-treated cows vs. respective control cows at each sampling time allowed to identify 35 DE-miRNAs, each one of them resulted differentially expressed (*p* < 0.05) at specific sampling times considered. Among these, the bta-miR-10167-3p was chosen for its potential use as diagnostic biomarkers for illicit rbST treatment, as it showed in general a consistent upregulation in rbST-treated cows at all sampling times considered, resulting statistically significant in the T3 and T5 groups (*p* < 0.05).

The qPCR analysis was therefore limited to only bta-miR-10167-3p rather than a multi-target qPCR array as those developed in other studies [23,43]. This choice was made firstly to include a larger number of field samples collected from healthy, well-managed cows and secondly to test the feasibility of a simple, cost-effective screening tool for future official residue control programs [20].

The qPCR analysis confirmed, as preliminarily shown by sequencing data, that mean expression levels of bta-miR-10167-3p in milk EV from rbST-treated dairy cows are higher in comparison to milk EV expression levels from control untreated animals (Figure 6). The increased expression of the tested miRNA in milk EV from rbST-treated cows became even higher when compared to mean expression levels found in tested field dairy cows (Figure 6). Taken together, these data suggest that bta-miR-10167-3p could have suitable application as diagnostic biomarkers for screening of suspected rbST misuse in dairy cows. Receiver operating characteristic (ROC) curves reported in Figure 7 shown indeed that expression levels of bta-miR-10167-3p (normalized on expression of bta-miR-29a, taken as endogenous control gene) could correlate with the rbST-treated status of dairy cows.

However, significant differences (*p* < 0.001) in the expression levels of bta-miR-10167-3p between the untreated rbST control group and field samples group suggest that further investigations on stability of this miRNA and its roles in milk EV are needed.

The currently available literature suggests that bta-miR-10167-3p plays a key role in bovine preadipocyte differentiation and proliferation, acting together with circular RNAs element like circADAMTS16 as an endogenous RNA network in the post-transcriptional translation of specific genes [44].

Another study on significant SNPs associated with milk production traits in dairy cows reported the presence of T cell factor 7 L1 gene (TCF7L1) among others relevant genetic target implied in such multi-trait phenotype [45]. The TCF7L1 is a genetic target for which bta-miR-10167-3p has shown important modulation effects in a recent study on bovine preadipocyte differentiation and apoptosis [46]. From the cited study, further gene ontology (GO) and Kyoto Encyclopedia of Genes and Genomes (KEGG) findings reported enriched molecular functions like cytoskeletal protein binding, vesicle and membrane organization, and specific enriched pathways like endocytosis, mTOR, and AMPK signaling. Taken together, these data suggest, as already stated, the need for further studies to better understand the role and the presence of significant upregulation of bta-miR-10167-3p levels in milk EVs from rbST-treated dairy cows.

## 4. Materials and Methods

### 4.1. Sample Selection

#### 4.1.1. Experimental Samples

A retrospective longitudinal study was developed on milk samples collected during an animal trial performed in Spain between 2016 and 2017 by Lamas and colleagues [23]. Briefly, in the original study nine Holstein cows in first or second lactation (age range: 1.5–4 years) were enrolled in long-term animal trial to study the effects of exogenous somatotropin administration. The animals were kept under conventional intensive farming regime, (non-access to pasture, premix diet, water ad libitum, etc.) as reported in original works from Lamas and colleagues [22,23]. They were therefore divided into two groups: a control group (n = 3) and rbST-treated group (n = 6). The rbST group was treated with 500 mg of rbST (Lactotropina^®^, Elanco^®^, Eli Lilly, Mexico) every 14 days, according to scheduling suggested by the manufacturer, for a total period of 6 months. At the time of the first rbST dose, selected animals were between 67 and 75 ± 4 days of lactation. From the original archival milk samples collected during the study (kept skimmed and refrigerated at −20 °C), a total of 63 samples from seven different time points were available:

- T0: 6 days before first rbST dose
- T1: 1 day after first rbST dose
- T2: 3 days after second rbST dose (i.e., 17 days after first rbST dose)
- T3: 7 days before fourth rbST dose (i.e., 35 days after first rbST dose)
- T4: 14 days before sixth rbST dose (i.e., 70 days after first rbST dose)
- T5: On the day of the sixth rbST dose (i.e., 84 days after first rbST dose)
- T6: 4 days after the sixth rbST dose (i.e., 88 days after first rbST dose)

Samplings distribution during the animal trial is further represented in Figure 1. During all milk sampling activities performed at the time of the original animal trial [23,28], routinary determinations for somatic cells count, fat, protein, lactose, and dry matter were performed using MilkoScanTM 7RM at the Galician Interprofessional Laboratory of Milk (LIGAL) using the PE/LIGAL/34 protocol, developed according to ISO/IEC 17025:2017.

After sequencing analysis on 63 samples belonging to reported sampling times (see Section 4.5), an additional experimental sample from a single cow belonging to rbST-treated group (two days after the first rbST injection) was added to the study (see blue arrow in Figure 1), as it was still available from the original archival sample batches. It was analyzed by qPCR, together with samples collected during field survey, as an external positive control sample (see Section 4.6).

#### 4.1.2. Field Samples and Milk Quality Evaluations

After experimental specimens’ collection and analysis, a field survey was then started to validate potential application of differentially expressed (DE) miRNA as rbST biomarkers.

Milk specimens were collected every 20–30 days from six Holstein bred, pluriparous, dairy cows, selected from farms located in the Piedmont region (north-western Italy), that apply common intensive livestock farming practices. Each cow was followed for seven months of lactation, respectively.

In order to limit potential preanalytical biases between archival milk samples and the field samples from the Italian survey, the same sampling procedures originally described by Lamas and colleagues were followed [23]. In depth, each milk sample was freshly collected on site and immediately transported to the laboratory in cold boxes. Upon arrival, samples were centrifuged and supernatant was separated and stored at −20 °C.

Besides analysis on skimmed milk specimens for EVs isolation and miRNA content profiling (see above), routinary composition analysis on raw milk were made to assess its quality and correct milking practices, with the aim to check for the presence of potential perturbations in miRNA profiles related to unexpected field conditions. Therefore, total proteins, fats, and lactose content were verified together with total and differential somatic cell counts and total bacteria count following standardized and validated methods (Reference methods: ISO 9622/IDF 141:2013; ISO 13366-2:2006/IDF 148-2:2006; UNI EN ISO 5764:2009).

### 4.2. EVs Isolation from Milk Samples

A total of 500 uL of skimmed milk was centrifuged at 16,000× *g* for 15 min at 4 °C (to remove residual fat, cell debris, and casein fraction) and 126 µL of Exoquick—TC (System Biosciences, Palo Alto, CA, USA) was added. After an overnight incubation, the mixture (milk/Exoquick) was centrifuge at 1500× *g* for 30 min at 4 °C and the resulting pellet was resuspended in 200 µL of PBS.

### 4.3. Nanoparticle Tracking Analysis (NTA)

The EVs pellet was resuspended in PBS. After a 1:1000 dilution EVs were visualized on the Nanosight LM10 instrument (Particle Characterization Laboratories, Novato, CA, USA). The particle size profile and concentration in the milk samples were evaluated by the NTA software (version 2.2, NanoSight).

### 4.4. Total RNA Purification

Resuspended EVs pellets (200 µL PBS) were mixed with 1 mL of QIAzol Lysis reagent (Qiagen, Hilden, Germany). RNAs were then purified using miRNeasy serum/plasma kit (Qiagen) in an automated way using the QIAcube instrument (Qiagen), avoiding operator-depending variability. Total RNAs was estimated quantitatively with Qubit™ microRNA Assay Kits (ThermoFisher Scientific, Waltham, MA, USA) and then stored at −80 °C until used.

### 4.5. Small RNA Sequencing Workflow

Small RNA libraries were prepared from samples collected on experimental animals (n = 63) using the NebNext Small RNA Library Preparation Kit (New England Biolabs, Ipswich, MA, USA) for miRNA and short non-coding RNAs sequencing according to manufacturer instructions, starting from 20 ng RNA and using 15 PCR cycles. Each library was analyzed with the High Sensitivity DNA chip (Agilent, Santa Clara, CA, USA) using Agilent 2100 Bioanalyzer. Libraries pools were concentrated with AMPure XP magnetic beads (Beckman Coulter, Brea, CA, USA) and eluted in 40 uL of nuclease free water. The eluate from each pool was loaded onto a 6% precast gel (EC6265BOX, ThermoFisher Scientific) and a ≈ 146 bp band was cut, purified with a 5 um filter fromQiaquick gel extraction kit (Qiagen), and quantified using the Qubit DNA HS (ThermoFisher Scientific). The final pool was run at the concentration of 1200 pM on the NextSeq 1000 sequencer (Illumina, San Diego, CA, USA) according to manufacturer instruction in 75 nts single end sequencing mode.

### 4.6. Biomarker Validation Study

A confirmatory analysis of DE miRNA identified by NGS sequencing was performed by quantitative real time PCR (qPCR) on all samples collected from experimental animals (n = 64) plus all samples collected from the six dairy cows enrolled in the field study (n = 70).

For the selection of endogenous miRNAs suitable for normalization of gene expression profiles, a preliminary stability evaluation was performed on 5 different micro RNA (bta-miR-29a, bta-miR-200a, bta-miR-26b, bta-miR-27b-3p, and bta-miR-30b-5p) previously described by Rahman et al., 2023 [47], using geNorm [48] and NormFinder [49] programs embedded in GenEx 6.1 software (MultiID, Gothenburg, Sweden).

Ten nanograms of total RNA from both field and experimental samples were retro transcribed into cDNA with TaqMan™ MicroRNA Reverse Transcription Kit (ThermoFisher Scientific), then amplified and analyzed on a Biorad CFX thermalcycler (Biorad, Hercules, CA, USA) with inventoried Taqman miRNA assay, specific for each selected miRNA (see Table 2), following manufacturer guidelines (ThermoFisher Scientific). Each sample was analyzed in duplicate for each target.

After the selection of the best endogenous control, all RNA samples were analyzed for relative expression calculations by ∆∆Cq method [50] to quantify the fold changes (FCs) of bta-miR-10167-3p (Table 2), resulted as the most relevant DE miRNA according to sequencing analysis (see results at Section 2.3).

### 4.7. Bioinformatics and Statistical Analysis

For the identification of differentially expressed (DE)-miRNAs from small-RNA sequencing workflow, all fastq files were analyzed with the miRNA pipeline included in docker4seq package [51], briefly consisting in a first step of reads filtering ad trimming (Cutadapt), fastq quality evaluation (Fastqc), mapping to miRBase haipins sequences (SHRiMP) followed by quantification of only the reads mapping on mature miRNAs (countOverlaps function from GenomicRanges package). Significance of relative expression values for each detected miRNA was then determined by two separate approaches:

- Detection and filtering out of all miRNAs perturbations linked to expected physiological variations, that are known to be present during different phases of lactation, by using Anova-like function from edgeR package. This analysis compared only untreated samples from each T1–T6 sampling times versus T0 sampling time.
- Differential analysis (DESeq2 package) for identification of DE-miRNAs potentially related to rbST exposure, by merging six separate rbST-treated vs. untreated comparisons, considering each sampling times (T1–T6). DE-miRNAs were defined by recorded fold changes (|log2FC| ≥1) and statistical significance (*p* < 0.05).

Heatmaps from the two DE-miRNA detection strategies listed above were generated with Morpheus software (https://software.broadinstitute.org/morpheus), set for row clustering by Euclidean distance and average linkage.

An additional principal component analysis (PCA) was performed on miRNA sequencing dataset to identify possible trends in the data or between rbST and control groups.

For gene expression data collected by quantitative Real Time PCR (qPCR), all Cq values were analyzed with GenEX software 6.1 (MultiID). Normal distribution of data was evaluated by Kolomogorov–Smirnov’s test. Comparisons between rbST-treated (T1–T6) vs. control groups (T1c–T6c) and between rbST-treated (T1–T6) vs. field samples group were assessed by Student’s *t*-test after checking normally distributed data. Significance in fold changes (FCs) from qPCR analysis was set at |log2FC| ≥ 1 with *p* < 0.05.

Finally, two receiver operator curves (ROC) were assessed to check the diagnostic potential of a candidate miRNA (bta-miR-10167-3p) to detect rbST illicit treatment by Prism software version 7 (GraphPad, La Jolla, CA, USA). The first ROC curve was calculated considering only rbST-untreated cows from the original animal trial as controls, and the second ROC curve was calculated considering field samples collected during the survey in local farms as controls.

## 5. Conclusions

The small RNA analysis strategy applied in our study allowed for the identification of candidate miRNA target perturbations that can be related to rbST treatment by filtering out the differential abundance of miRNA that are known to exhibit expected and considerable changes during lactation, without being significantly related to the studied illicit treatment. The successful analysis on archival milk samples, available from a previous animal trial, confirm nowadays the importance of biobank management and its contribution to the principles of 3R (reduction, replacement, and refinement) in experiments involving animals, as reiterated by EU Directive 2010/63. Indeed, these crucial aspects have also been observed in other omics-based studies regarding illicit administration of growth promoters, based for example on retrospective analysis of formalin-fixed paraffin-embedded (FFPE) tissues [43].

Among the 35 DE-miRNAs identified by applied approach, the bta-miR-10167-3p resulted as constantly overexpressed in rbST-treated dairy cows, at least across the 4 months of lactation considered in the present work.

Further studies to verify the diagnostic potential of bta-miR-10167-3p and the other DE miRNAs are needed but, in any case, the preliminary results by field screening on milk samples collected from Italian farms seem to confirm the reliability of applied workflows for novel biomarkers discovery and, consequently, our findings.

In conclusion, to implement these preliminary results into real field conditions, an extensive study to assess reference/basal expression levels of bta-miR-10167-3p in a larger dairy cows’ population will need to be set. Moreover, by considering both individual and bulk milk sampling strategies, it will be possible to clarify the potential use of this miRNA as an in vivo non-invasive screening test to help contrast illicit rbST treatment in dairy cows.

## Figures and Tables

**Table 2:** Taqman assays used for normalization and relative expression analysis of the bta-miR-10167-3p.

miRNA	miRbase Accession	Sequence	Taqman Assay ID
bta-miR-29a	MIMAT0003518	CUAGCACCAUCUGAAAUCGGUUA	007600
bta-miR-200a	MIMAT0003822	UAACACUGUCUGGUAACGAUGUU	006209
bta-miR-26b	MIMAT0003531	UUCAAGUAAUUCAGGAUAGGUU	000406
bta-miR-27b	MIMAT0003546	UUCACAGUGGCUAAGUUCUGC	000409
bta-miR-30b-5p	MIMAT0003547	UGUAAACAUCCUACACUCAGCU	000602
bta-miR-10167-3p	MIMAT0040914	CGGGUGGUCGGGGCGGGUCAG	CS7FHME

## Data Availability

The data presented in this study are available on request from the corresponding author.

## Supplementary Materials

The following supporting information can be downloaded at: https://www.mdpi.com/article/10.3390/ijms26062437/s1.
