# Supplementary material for: miRNome Characterization of Milk-Derived Extracellular Vesicles in Recombinant Somatotropin-Treated Dairy Cows
Source: Int J Mol Sci. 2025 Mar 8;26(6):2437. doi: 10.3390/ijms26062437 (PMC11941771; doi:10.3390/ijms26062437)
Supplement: Supplementary file 1 [file ijms-26-02437-s001.zip › Supplementary Material S1.pdf]

Supplementary Material S1: Examples of NanoSight profiles of EVs isolated from skimmed milk samples collected during field survey started in 2022 (EV1) and archival samples coming from animal trial, performed by Lamas and coauthors in 2016-2017 (EV4-EV5), to check absence of adverse effects on EVs due to prolonged storage.

## NANOSIGHT

**EV1 2022-11-23 15-23-35**

Field sample

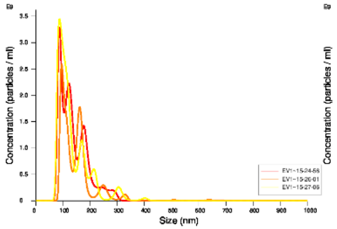


FTLA Concentration / Size graph for Experiment:
EV1 2022-11-23 15-23-35

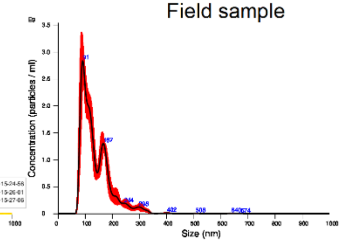


Averaged FTLA Concentration / Size for Experiment:
EV1 2022-11-23 15-23-35
Error bars indicate + / -1 standard error of the mean

**Included Files**

EV1 2022-11-23 15-24-56
EV1 2022-11-23 15-26-01
EV1 2022-11-23 15-27-06

**Details**

| | |
|---|---|
| NTA Version: | NTA 3.2 Dev Build 3.2.16 |
| Script Used: | SOP Standard Measurement 03-23-35PM 23~ |
| Time Captured: | 15:23:35 23/11/2022 |
| Operator: | |
| Pre-treatment: | |
| Sample Name: | |
| Diluent: | |
| Remarks: | |

**Capture Settings**

| | |
|---|---|
| Camera Type: | sCMOS |
| Laser Type: | Blue488 |
| Camera Level: | 15 |
| Slider Shutter: | 1206 |
| Slider Gain: | 366 |
| FPS | 25.0 |
| Number of Frames: | 1498 |
| Temperature: | 23.1 - 23.3 ºC |
| Viscosity: | (Water) 0.925 - 0.928 cP |
| Dilution factor: | 5 x 10e2 |
| Syringe Pump Speed: | 30 |

**Analysis Settings**

| | |
|---|---|
| Detect Threshold: | 3 |
| Blur Size: | Auto |
| Max Jump Distance: | Auto: 11.6 - 12.8 pix |

**Results**

Stats: Merged Data

| | |
|---|---|
| Mean: | 138.0 nm |
| Mode: | 90.7 nm |
| SD: | 57.6 nm |
| D10: | 84.3 nm |
| D50: | 119.5 nm |
| D90: | 207.6 nm |

Stats: Mean +/- Standard Error

| | |
|---|---|
| Mean: | 138.7 +/- 3.5 nm |
| Mode: | 89.3 +/- 2.7 nm |
| SD: | 57.7 +/- 1.8 nm |
| D10: | 85.4 +/- 3.1 nm |
| D50: | 121.6 +/- 5.3 nm |
| D90: | 210.7 +/- 4.3 nm |
| Concentration (Upgrade): | 2.02e+011 +/- 2.38e+010 particles/ml |
| | 55.4 +/- 6.1 particles/frame |
| | 66.2 +/- 6.1 centres/frame |

Concentration measurements may require some caution due to noise
See summary file for more info

## NANOSIGHT

**EV4 2022-11-23 15-34-27**

rbST treated sample

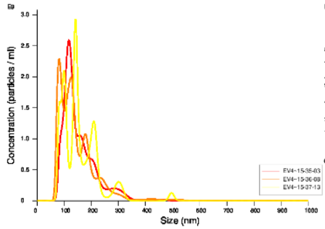


FTLA Concentration / Size graph for Experiment:
EV4 2022-11-23 15-34-27

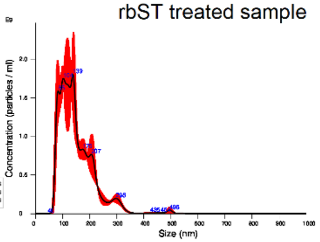


Averaged FTLA Concentration / Size for Experiment:
EV4 2022-11-23 15-34-27
Error bars indicate + / -1 standard error of the mean

**Included Files**

EV4 2022-11-23 15-35-03
EV4 2022-11-23 15-36-08
EV4 2022-11-23 15-37-13

**Details**

| | |
|---|---|
| NTA Version: | NTA 3.2 Dev Build 3.2.16 |
| Script Used: | SOP Standard Measurement 03-34-27PM 23~ |
| Time Captured: | 15:34:27 23/11/2022 |
| Operator: | |
| Pre-treatment: | |
| Sample Name: | |
| Diluent: | |
| Remarks: | |

**Capture Settings**

| | |
|---|---|
| Camera Type: | sCMOS |
| Laser Type: | Blue488 |
| Camera Level: | 15 |
| Slider Shutter: | 1206 |
| Slider Gain: | 366 |
| FPS | 25.0 |
| Number of Frames: | 1498 |
| Temperature: | 23.2 - 23.3 ºC |
| Viscosity: | (Water) 0.925 - 0.927 cP |
| Dilution factor: | 5 x 10e2 |
| Syringe Pump Speed: | 30 |

**Analysis Settings**

| | |
|---|---|
| Detect Threshold: | 3 |
| Blur Size: | Auto |
| Max Jump Distance: | Auto: 11.8 - 13.2 pix |

**Results**

Stats: Merged Data

| | |
|---|---|
| Mean: | 151.3 nm |
| Mode: | 138.9 nm |
| SD: | 66.0 nm |
| D10: | 84.7 nm |
| D50: | 134.8 nm |
| D90: | 227.6 nm |

Stats: Mean +/- Standard Error

| | |
|---|---|
| Mean: | 151.4 +/- 4.3 nm |
| Mode: | 113.2 +/- 17.5 nm |
| SD: | 65.5 +/- 3.2 nm |
| D10: | 86.4 +/- 4.7 nm |
| D50: | 133.3 +/- 4.8 nm |
| D90: | 227.9 +/- 1.6 nm |
| Concentration (Upgrade): | 2.08e+011 +/- 4.00e+009 particles/ml |
| | 59.6 +/- 1.4 particles/frame |
| | 80.1 +/- 3.2 centres/frame |

Concentration measurements may require some caution due to noise
See summary file for more info

## NANOSIGHT

**EV5 2022-11-23 15-44-50**

rbST untreated sample

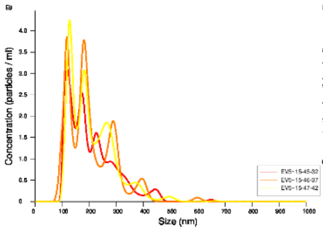


FTLA Concentration / Size graph for Experiment:
EV5 2022-11-23 15-44-50

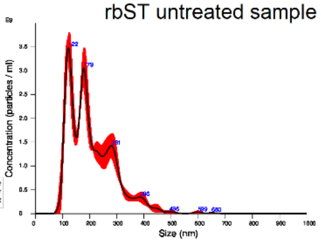


Averaged FTLA Concentration / Size for Experiment:
EV5 2022-11-23 15-44-50
Error bars indicate + / -1 standard error of the mean

**Included Files**

EV5 2022-11-23 15-45-32
EV5 2022-11-23 15-46-37
EV5 2022-11-23 15-47-42

**Details**

| | |
|---|---|
| NTA Version: | NTA 3.2 Dev Build 3.2.16 |
| Script Used: | SOP Standard Measurement 03-44-50PM 23~ |
| Time Captured: | 15:44:50 23/11/2022 |
| Operator: | |
| Pre-treatment: | |
| Sample Name: | |
| Diluent: | |
| Remarks: | |

**Capture Settings**

| | |
|---|---|
| Camera Type: | sCMOS |
| Laser Type: | Blue488 |
| Camera Level: | 13 |
| Slider Shutter: | 1232 |
| Slider Gain: | 219 |
| FPS | 25.0 |
| Number of Frames: | 1498 |
| Temperature: | 23.3 - 23.4 ºC |
| Viscosity: | (Water) 0.922 - 0.925 cP |
| Dilution factor: | 5 x 10e2 |
| Syringe Pump Speed: | 30 |

**Analysis Settings**

| | |
|---|---|
| Detect Threshold: | 3 |
| Blur Size: | Auto |
| Max Jump Distance: | Auto: 10.1 - 11.3 pix |

**Results**

Stats: Merged Data

| | |
|---|---|
| Mean: | 206.4 nm |
| Mode: | 121.7 nm |
| SD: | 85.5 nm |
| D10: | 114.5 nm |
| D50: | 184.8 nm |
| D90: | 315.1 nm |

Stats: Mean +/- Standard Error

| | |
|---|---|
| Mean: | 206.4 +/- 1.7 nm |
| Mode: | 120.7 +/- 3.3 nm |
| SD: | 85.6 +/- 1.4 nm |
| D10: | 114.6 +/- 3.2 nm |
| D50: | 184.6 +/- 2.1 nm |
| D90: | 315.8 +/- 5.0 nm |
| Concentration (Upgrade): | 4.44e+011 +/- 2.45e+010 particles/ml |
| | 125.8 +/- 6.6 particles/frame |
| | 154.5 +/- 6.4 centres/frame |

Concentration measurements may require some caution due to noise
See summary file for more info

## NANOSIGHT

**EV1 2022-11-23 15-23-35**

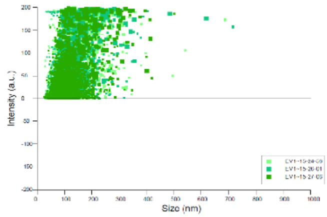


Intensity / Size graph for Experiment:
EV1 2022-11-23 15-23-35

## NANOSIGHT

**EV4 2022-11-23 15-34-27**

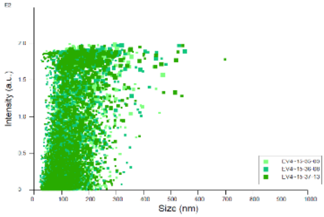


Intensity / Size graph for Experiment:
EV4 2022-11-23 15-34-27

## NANOSIGHT

**EV5 2022-11-23 15-44-50**

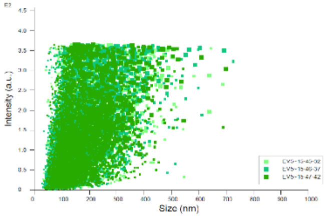


Intensity / Size graph for Experiment:
EV5 2022-11-23 15-44-50
