# Supplementary material for: miRNome Characterization of Milk-Derived Extracellular Vesicles in Recombinant Somatotropin-Treated Dairy Cows
Source: Int J Mol Sci. 2025 Mar 8;26(6):2437. doi: 10.3390/ijms26062437 (PMC11941771; doi:10.3390/ijms26062437)
Supplement: Supplementary file 1 [file ijms-26-02437-s001.zip › Supplementary Material S2_mods.pdf]

ANALYSIS	TCB	FAT	PROTEIN	LACTOSE	TOTAL SOMATIC CELL COUNT	DSCC	date
UNIT	*1000CFU/ml	g/100g	g/100g	g/100g	*1000cell/ml	%(n°lymphocytes and PMN)	
cow 1	13	3.82	3.94	4.91	38	0	2022-11-21
cow 2	3	3.26	3.16	4.95	143	75	2022-11-21
cow 3	16	4.16	3.26	5.09	50	63	2022-11-21
cow 4	3	3.4	4.17	4.85	20	0	2022-11-21
cow 5	5	3.64	3.2	4.91	21	0	2022-11-21
cow 6	3	3.64	3.07	4.83	9	0	2022-11-21
cow 1	12	4.12	3.84	4.89	20	0	2022-12-05
cow 2	20	4.01	3.18	4.98	26	0	2022-12-05
cow 3	13	4.5	3.34	5.14	18	0	2022-12-05
cow 4	5	4.18	4.31	4.79	14	0	2022-12-05
cow 5	8	4.24	3.95	5.11	23	0	2022-12-05
cow 6	7	3.92	3.85	4.85	6	0	2022-12-05
cow 1	9	3.885	3.04	4.9	32	0	2022-12-27
cow 2	5	4.27	3.15	4.98	12	0	2022-12-27
cow 3	22	4.54	3.42	5	29	0	2022-12-27
cow 4	2	4.1	4.45	4.85	27	0	2022-12-27
cow 5	7	3.79	3.01	4.99	17	0	2022-12-27
cow 6	8	4.31	3.01	4.92	13	0	2022-12-27
cow 1	6	3.9	3.03	4.81	55	74	2023-01-11
cow 2	5	3.64	3.14	4.96	8	0	2023-01-11
cow 3	6	4.44	3.64	5.15	4	0	2023-01-11
cow 4	3	3.19	4.3	4.61	43	0	2023-01-11
cow 5	6	3.95	3.76	5.04	50	74	2023-01-11
cow 6	2	4.09	3.98	4.9	6	0	2023-01-11
cow 1	8	4.14	3.1	4.83	49	0	2023-01-26
cow 2	7	3.84	3.41	5	4	0	2023-01-26
cow 3	24	4.46	3.91	5	42	0	2023-01-26
cow 4	3	3.04	4.42	4.8	31	0	2023-01-26
cow 5	5	4.16	3.22	4.91	37	0	2023-01-26
cow 6	4	4.15	3.15	4.92	6	0	2023-01-26
cow 1	12	4.37	3.25	4.72	20	0	2023-02-10

cow 2	17	4.27	3.27	4.98	10	0	2023-02-10
cow 3	13	3.04	3.71	5.04	6	0	2023-02-10
cow 4	10	4.67	4.49	4.74	70	67	2023-02-10
cow 5	11	3.64	3.26	4.8	32	0	2023-02-10
cow 6	11	3.41	3.26	4.9	12	0	2023-02-10
cow 1	12	4.37	3.25	4.72	20	0	2023-02-27
cow 2	17	4.27	3.27	4.98	10	0	2023-02-27
cow 3	13	3.04	3.71	5.04	6	0	2023-02-27
cow 4	10	4.67	4.49	4.74	70	67	2023-02-27
cow 5	11	3.64	3.26	4.8	32	0	2023-02-27
cow 6	11	3.41	3.26	4.9	12	0	2023-02-27
cow 1	6	4.61	3.18	4.81	67	64	2023-03-13
cow 2	3	3.71	3.65	4.88	22	0	2023-03-13
cow 3	6	4.42	3.83	4.78	1153	70	2023-03-13
cow 4	2	4.68	4.32	4.49	128	60	2023-03-13
cow 5	15	3.66	3.29	4.81	69	71	2023-03-13
cow 6	12	3.72	3.19	4.81	9	0	2023-03-13
cow 1	1	3.03	3.36	4.77	29	0	2023-03-29
cow 2							
cow 3	1	4.85	4.11	5.00	3	0	2023-03-29
cow 4	2	4.89	4.29	4.72	23	0	2023-03-29
cow 5	3	3.35	3.36	4.87	20	0	2023-03-29
cow 6	2	3.70	3.17	4.88	6	0	2023-03-29
cow 1	7	3.84	3.27	4.66	78	68	2023-04-28
cow 2	14	4.03	3.82	5.03	20	0	2023-04-28
cow 3	9	5.00	4.55	3.70	717	77	2023-04-28
cow 4	3	4.54	3.26	4.95	24	0	2023-04-28
cow 5	3	4.61	3.06	4.90	16	0	2023-04-28
cow 6							
cow 1	7	4.34	3.36	4.77	134	75	2023-05-29
cow 2							
cow 3	3	4.29	4.04	4.98	8	0	2023-05-29
cow 4	4	4.66	4.68	4.55	32	0	2023-05-29

cow 5	5	4.12	3.53	4.95	25	0	2023-05-29
cow 6	6	3.74	3.19	4.87	9	0	2023-05-29
cow 7	9	3.70	3.56	4.74	151	72	2023-05-29
cow 1	7	3.72	3,43	4,66	155	64	2023-06-26
cow 2							
cow 3	2	4.06	3,99	5,09	5	0	2023-06-26
cow 4	2	3.28	5,08	4,31	28	0	2023-06-26
cow 5	2	3.76	3,31	4,95	43	0	2023-06-26
cow 6	1	3.55	3,23	4,86	10	0	2023-06-26
cow 7	63	4,01	3,54	4,65	329	77	2023-06-26
