# Supplementary material for: miRNome Characterization of Milk-Derived Extracellular Vesicles in Recombinant Somatotropin-Treated Dairy Cows
Source: Int J Mol Sci. 2025 Mar 8;26(6):2437. doi: 10.3390/ijms26062437 (PMC11941771; doi:10.3390/ijms26062437)
Supplement: Supplementary file 1 [file ijms-26-02437-s001.zip › Supplementary Material S3.pdf]

Red cluster	miRBase accession	Blue cluster	miRBase accession	Green Cluster	miRBase accession	Yellow cluster	miRBase accession	Purple cluster	miRBase accession
bta-miR-140	MIMAT0003789	bta-miR-2372	MIMAT0011917	bta-miR-2413	MIMAT0011975	bta-miR-200c	MIMAT0003823	bta-miR-133a-2	MI0009732
bta-miR-133a-2	MI0009732	bta-miR-2484	MIMAT0012077	bta-miR-669	MIMAT0013838	bta-miR-200a	MIMAT0003822	bta-miR-2467-3p	MIMAT0012057
bta-miR-376d	MIMAT0010199	bta-miR-2409	MIMAT0011967	bta-miR-2344	MIMAT0011879	bta-miR-3596	MIMAT0016940	bta-miR-2290	MIMAT0011798
bta-miR-12053	MIMAT0046760	bta-miR-1287	MIMAT0009971	bta-miR-1260b	MIMAT0024568	bta-miR-320a-1	MI0012211	bta-miR-2888-1	MI0013062
bta-miR-2284w	MIMAT0017393	bta-miR-2341	MIMAT0011876	bta-miR-323b	MIMAT0040935	bta-miR-320a-2	MI0004748	bta-miR-744	MIMAT0009369
bta-miR-154b	MIMAT0025547	bta-miR-488	MIMAT0009330	bta-miR-877	MIMAT0009381	bta-miR-148d	MIMAT0046670	bta-miR-2418	MIMAT0011982
bta-miR-381	MIMAT0009307	bta-miR-12060	MIMAT0046767	bta-miR-2478	MIMAT0012070	bta-miR-148a	MIMAT0003522	bta-miR-2394	MIMAT0011947
bta-miR-2422	MIMAT0011989	bta-miR-11995	MIMAT0046648	bta-miR-12029	MIMAT0046722	bta-miR-30a-5p	MIMAT0003841	bta-miR-769	MIMAT0009376
bta-miR-2448-3p	MIMAT0012026	bta-miR-2458	MIMAT0012043	bta-miR-2897	MIMAT0013855	bta-let-7f-1	MI0005062	bta-miR-432	MIMAT0009317
bta-miR-2381	MIMAT0011929	bta-miR-2900	MIMAT0013858	bta-miR-497	MIMAT0004343	bta-let-7a-3	MI0005452	bta-miR-2388-5p	MIMAT0011940
bta-miR-1307	MIMAT0009969	bta-miR-7857-2	MI0032928	bta-miR-375	MIMAT0009303	bta-let-7a-2	MI0005451	bta-miR-34b	MIMAT0003549
bta-miR-2378	MIMAT0011924	bta-miR-2462	MIMAT0012049	bta-miR-2382-3p	MIMAT0011931	bta-let-7a-1	MI0005057	bta-miR-219b-3p	MIMAT0040926
bta-miR-2477	MIMAT0012069	bta-miR-1251	MIMAT0009963	bta-miR-12032	MIMAT0046725	bta-miR-2284x	MIMAT0017395	bta-miR-219-1	MI0009781
bta-miR-12058	MIMAT0046765	bta-miR-11981	MIMAT0046370	bta-miR-18a	MIMAT0003526	bta-miR-1307	MIMAT0009969	bta-miR-2311	MIMAT0011826
bta-miR-6520	MIMAT0025538	bta-miR-11983	MIMAT0046376	bta-miR-12012	MIMAT0046680	bta-let-7c	MIMAT0004332	bta-miR-11986c	MIMAT0046638
bta-miR-658	MIMAT0009362	bta-miR-2393	MIMAT0011946	bta-miR-378d	MIMAT0036972	bta-let-7e	MIMAT0004333	bta-miR-6532	MIMAT0025568
bta-miR-544b-1	MI0009860	bta-miR-1281	MIMAT0009962	bta-miR-11972	MIMAT0046341	bta-miR-374a	MIMAT0004342	bta-miR-2377	MIMAT0011923
bta-miR-11978	MIMAT0046364	bta-miR-3533	MIMAT0036975	bta-miR-4449	MIMAT0036973	bta-miR-200b	MIMAT0003842	bta-miR-12063	MIMAT0046770
bta-miR-12027	MIMAT0046720	bta-miR-2304	MIMAT0011816	bta-miR-10177	MIMAT0040932	bta-miR-186	MIMAT0003818	bta-miR-3956	MIMAT0025544
bta-miR-205	MIMAT0003545	bta-miR-31	MIMAT0003548	bta-miR-11972	MIMAT0046341	bta-let-7b	MIMAT0004331	bta-miR-2309	MIMAT0011821
bta-miR-2284h-3p	MIMAT0012020	bta-miR-483	MIMAT0009327	bta-miR-11973	MIMAT0046349	bta-miR-27a-3p	MIMAT0003532	bta-miR-1721	MIMAT0011849
bta-miR-487b	MIMAT0003847	bta-miR-19a	MIMAT0004336	bta-miR-1224	MIMAT0009944	bta-miR-30e-5p	MIMAT0003799	bta-miR-7180	MIMAT0046626
bta-miR-2307	MIMAT0011819	bta-miR-12042	MIMAT0046739	bta-miR-2898	MIMAT0013856	bta-miR-148d	MIMAT0046670	bta-miR-2483-5p	MIMAT0012075
bta-miR-92a-1	MI0009905	bta-miR-2428	MIMAT0011998	bta-miR-2887-2	MI0013061	bta-miR-200a	MIMAT0003822	bta-miR-12010	MIMAT0046678
bta-miR-11974	MIMAT0046353			bta-miR-2887-1	MI0013060	bta-miR-660	MIMAT0004344	bta-miR-2285cf	MIMAT0046663
bta-miR-2353	MIMAT0011889			bta-miR-2890	MIMAT0013848	bta-miR-125b-1	MI0004753	bta-miR-2284p	MIMAT0011885
bta-miR-1301	MIMAT0009958			bta-miR-2892	MIMAT0013850	bta-miR-125b-2	MI0005457	bta-miR-2311	MIMAT0011826
bta-miR-2285n-6	MI0022311			bta-miR-11976-2	MI0038181	bta-miR-429	MIMAT0009315	bta-miR-1271	MIMAT0009975
bta-miR-502a-1	MI0009852			bta-miR-11976-1	MI0038180	bta-miR-192	MIMAT0003820	bta-miR-2285d	MIMAT0011815
bta-miR-2472	MIMAT0012063			bta-miR-12034	MIMAT0046727	bta-miR-181a-1	MI0010484	bta-miR-2486-5p	MIMAT0012081
bta-miR-11997	MIMAT0046653			bta-miR-2903	MIMAT0013861	bta-miR-182	MIMAT0009244	bta-miR-2284k	MIMAT0011907
bta-miR-488	MIMAT0009330			bta-miR-12030	MIMAT0046723	bta-miR-181a-2	MI0004757	bta-miR-2485	MIMAT0012079
bta-miR-431	MIMAT0009316			bta-miR-2360	MIMAT0011898	bta-miR-151-3p	MIMAT0003524	bta-miR-6526-2	MI0022314
bta-miR-7-1	MI0010471			bta-miR-2368-3p	MIMAT0011912	bta-miR-30f	MIMAT0009282	bta-miR-11997	MIMAT0046653
bta-miR-584-8	MI0010453			bta-miR-204	MIMAT0004338	bta-miR-6529b	MIMAT0029947	bta-miR-2285as-3	MI0038163
bta-miR-562	MIMAT0009349			bta-miR-2889	MIMAT0013847	bta-miR-6529a	MIMAT0025565	bta-miR-216b	MIMAT0009266
bta-miR-12050	MIMAT0046753					bta-miR-340	MIMAT0009296	bta-miR-2306	MIMAT0011818

Red cluster	miRBase accession
bta-miR-10163-3p	MIMAT0040910
bta-miR-1301	MIMAT0009958
bta-miR-206	MIMAT0009260
bta-miR-12039	MIMAT0046734
bta-miR-199b	MIMAT0003821
bta-miR-2285s	MIMAT0025576
bta-miR-2315	MIMAT0011835
bta-miR-2450d	MIMAT0036977
bta-miR-182	MIMAT0009244
bta-miR-105b	MIMAT0009216
bta-miR-574	MIMAT0024577
bta-miR-6518	MIMAT0025536
bta-miR-2427	MIMAT0011997
bta-miR-10178-5p	MIMAT0040933
bta-miR-2326	MIMAT0011855
bta-miR-12061	MIMAT0046768
bta-miR-12059	MIMAT0046766
bta-miR-584-4	MI0009870
bta-miR-10176-5p	MIMAT0040931
bta-miR-2285o-5	MI0022307
bta-miR-7860	MIMAT0030439
bta-miR-2450b	MIMAT0012029
bta-miR-6530	MIMAT0025566
bta-miR-544b-2	MI0009861
bta-miR-193b	MIMAT0009253
bta-miR-2379	MIMAT0011925
bta-miR-133a-1	MI0009733
bta-miR-12004	MIMAT0046664
bta-miR-381	MIMAT0009307
bta-miR-10b	MIMAT0003839
bta-miR-197	MIMAT0009257
bta-miR-19b-2	MI0012209
bta-miR-2285ac	MIMAT0030440
bta-miR-126-3p	MIMAT0003540

Yellow cluster	miRBase accession
bta-miR-155	MIMAT0009241
bta-miR-22-5p	MIMAT0003826
bta-miR-21-5p	MIMAT0003528
bta-miR-26a-1	MI0009784
bta-miR-26c	MIMAT0016938
bta-miR-26a-2	MI0004731
bta-miR-26b	MIMAT0003531
bta-miR-3600	MIMAT0016932
bta-miR-22-3p	MIMAT0012536
bta-miR-146b	MIMAT0009235

Purple cluster	miRBase accession
bta-miR-2292	MIMAT0011800
bta-miR-211	MIMAT0009263
bta-miR-449c	MIMAT0009322
bta-miR-1306	MIMAT0009974
